# A new structural arrangement in proteins involving lysine NH_3_^+^ group and carbonyl

**DOI:** 10.1038/s41598-017-16584-y

**Published:** 2017-11-27

**Authors:** Olga N. Rogacheva, Sergei A. Izmailov, Lyudmila V. Slipchenko, Nikolai R. Skrynnikov

**Affiliations:** 1 Laboratory of Biomolecular NMR, St. Petersburg State University, St. Petersburg, 199034 Russia; 2 Department of Chemistry, Purdue University, West Lafayette, IN 47907 USA; 3 Department of General Pathology and Pathophysiology, Institute of Experimental Medicine, St. Petersburg, 197376 Russia

## Abstract

Screening of the Protein Data Bank led to identification of a recurring structural motif where lysine NH_3_^+^ group interacts with backbone carbonyl. This interaction is characterized by linear atom arrangement, with carbonyl O atom positioned on the three-fold symmetry axis of the NH_3_^+^ group (angle C^ε^-N^ζ^-O close to 180°, distance N^ζ^-O ca. 2.7-3.0 Å). Typically, this linear arrangement coexists with three regular hydrogen bonds formed by lysine NH_3_^+^ group (angle C^ε^-N^ζ^-*acceptor atom* close to 109°, distance N^ζ^-*acceptor atom* ca. 2.7-3.0 Å). Our DFT calculations using polarizable continuum environment suggest that this newly identified linear interaction makes an appreciable contribution to protein’s energy balance, up to 2 kcal/mol. In the context of protein structure, linear interactions play a role in capping the C-termini of α-helices and 3_10_-helices. Of note, linear interaction involving conserved lysine is consistently found in the P-loop of numerous NTPase domains, where it stabilizes the substrate-binding conformation of the P-loop. Linear interaction NH_3_^+^ – carbonyl represents an interesting example of ion-dipole interactions that has so far received little attention compared to ion-ion interactions (salt bridges) and dipole-dipole interactions (hydrogen bonds), but nevertheless represents a distinctive element of protein architecture.

## Introduction

**Figure 1:**
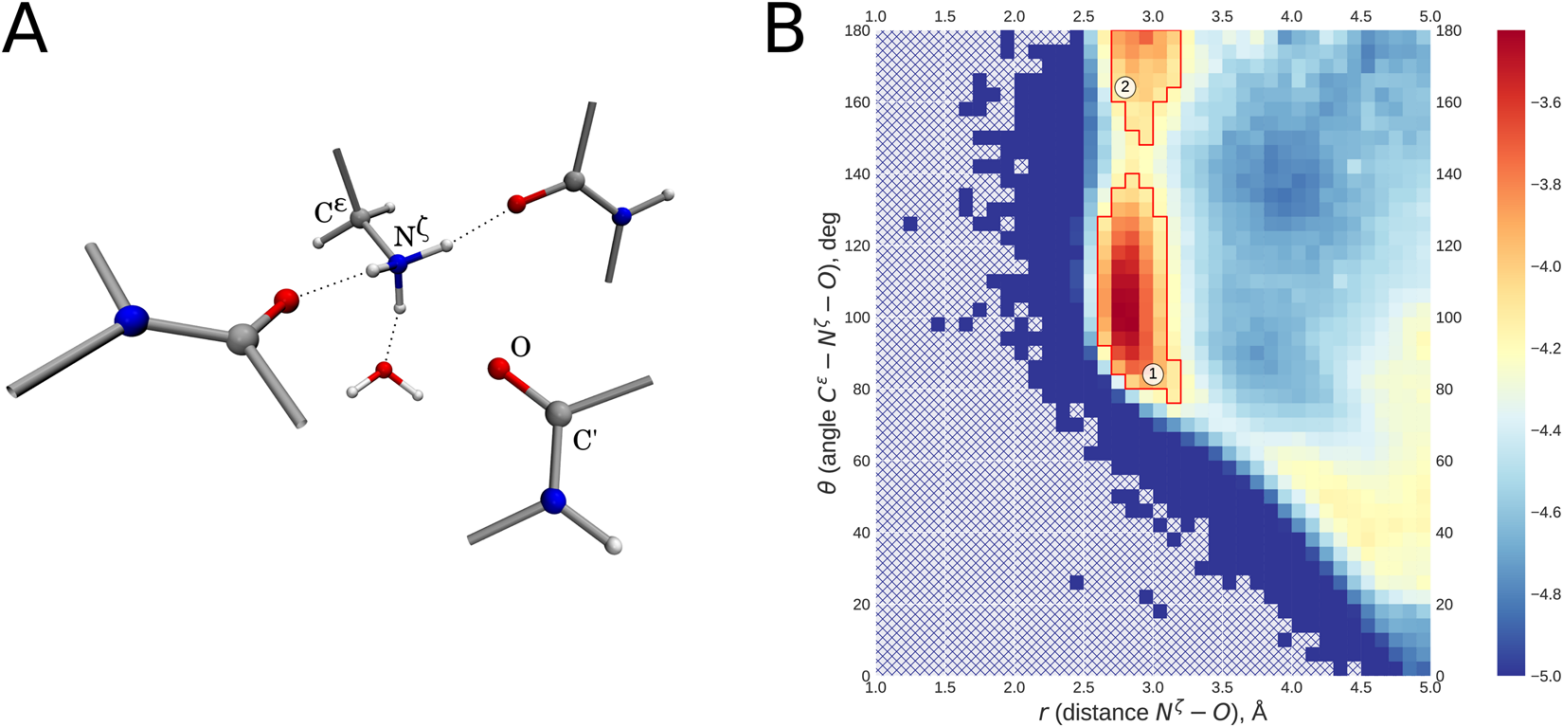
(**A**) Example of the lysine NH_3_^+^ – carbonyl linear arrangement as extracted from the crystal structure PDB ID 4RLZ^45^ (resolution 1.19 Å, *R*_*free*_ = 0.160). NH_3_^+^ group additionally forms hydrogen bonds to two other carbonyl sites and crystallographic water (shown by dashed lines). (**B**) Map illustrating relative positioning of the lysine NH_3_^+^ and proximal carbonyl groups based on comprehensive analysis of high- and medium-resolution x-ray structures in the Protein Data Bank. Shown in the map is the phase-space density *ρ*(*r*, *θ*), i.e. the number of lysine/carbonyl pairs that fall in a given square (pixel) divided by the total number of such pairs in the map and further divided by the volume of space corresponding to this pixel, $\Omega =2\pi {r}^{2}\,\sin \,\theta \,{\rm{\Delta }}r\,{\rm{\Delta }}\theta$. Note that the volume Ω is related to configurational entropy, $S=R\,\mathrm{ln}\,\Omega$, and therefore *ρ*(*r*, *θ*) reflects the enthalpy part of the free energy (as it should be for canonical ensemble in condensed phase)^46^. Color scale is logarithmic as indicated in the color bar on the side. Red contours around clusters 1 and 2 are drawn with the boundary $\mathrm{log}\,\rho =-4.14$. Shown in Fig. S1 are the analogues of this map using a more stringent selection of PDB structures limited to (*i*) very high resolution x-ray structures and/or (*ii*) unique structures excluding identical or highly similar proteins.

Regular hydrogen bonds between lysine charged ε-ammonium group and uncharged acceptors, as well as salt bridges between lysine and anionic residues, are well documented in the literature^1–4^. Yet inspection of the Protein Data Bank (PDB) finds many examples of an unusual interaction between lysine NH_3_^+^ and backbone carbonyl group, which is clearly different from a hydrogen bond. One example of such interaction is illustrated in Fig. 1A. Characteristically, carbonyl oxygen atom is positioned on the symmetry axis of NH_3_^+^ group, equidistant from the three ammonium protons. The angle C^ε^-N^ζ^-O in this arrangement is close to 180°, in contrast to hydrogen bonds where the corresponding angle is near 109°. Note also that this linear arrangement co-exists with three conventional hydrogen bonds centered on NH_3_^+^ group and directed toward other protein sites or toward crystallographic water (indicated by dashed lines in Fig. 1A).

In order to determine how typical this arrangement is, we have screened the Protein Data Bank for NH_3_^+^ – carbonyl pairs. Only high- and medium-resolution x-ray structures have been included in the analysis, resulting in a set of 47,388 unique structures (see Methods). The statistics are presented in Fig. 1B in a form of a map showing the density of states as a function of N^ζ^-O distance (*r*) and C^ε^-N^ζ^-O angle (*θ*). In this map we observe the dominant cluster, corresponding to a regular NH_3_^+^ – carbonyl hydrogen bond, which is centered at 2.8 Å, ca. 109° (cluster 1). At the same time we observe a distinct additional cluster (cluster 2), which corresponds to configurations illustrated in Fig. 1A with *θ* approaching 180° and the distance of ca. 2.9 Å (essentially the same distance as in hydrogen bonds). The number of such linear configurations is significant: within the red contour line delimiting cluster 2 we find 11,719 such examples. For comparison, cluster 1 contains 208,708 instances of hydrogen bonds originating on lysine NH_3_^+^ groups. To put these numbers into perspective, bear in mind that each NH_3_^+^ group can simultaneously form three hydrogen bonds, but only one linear interaction to carbonyl.

While PDB contains multiple examples of the linear arrangement involving NH_3_^+^ group and carbonyl, it is also desirable to present “true negative” controls. To this end, we have compiled analogous (*r*, *θ*) density maps for the pairs (*i*) NH_3_^+^ – backbone amide NH and (*ii*) NH_3_^+^ – side-chain methylene groups. The results clearly demonstrate that the cluster 2, corresponding to the linear interaction, is non-existent in both cases, see Fig. S2. This observation confirms that the linear placement of NH_3_^+^ and carbonyl is not an energy-neutral artefact of protein packing, but rather a meaningful interaction.

**Figure 2:**
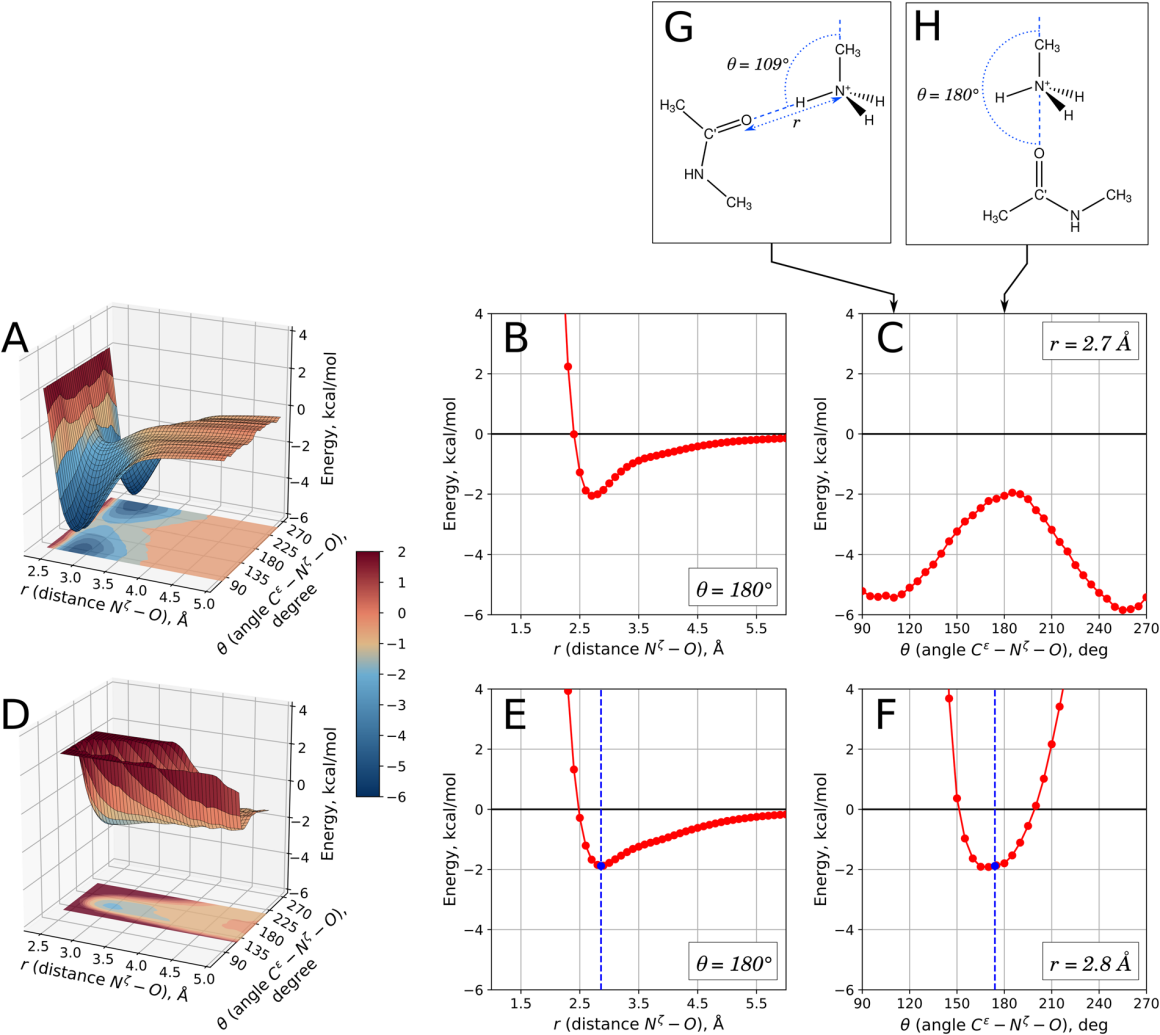
DFT energy scans for two models representative of the linear interaction between lysine NH_3_^+^ group and carbonyl. (**A**–**C**) Calculations using 2-molecule model consisting of methylammonium ion and N-methylacetamide. The model is illustrated in panels G (hydrogen bond arrangement) and H (linear arrangement). Note that the minimum at *θ* = 109° in panel C offers a more accurate estimation of energy for NH_3_^+^ – carbonyl hydrogen bond than symmetric minimum at *θ* = 360°–109° because the latter is additionally enhanced by a favorable packing of the methyl groups. (**D**–**F**) Calculations using 5-molecule model, based on PDB ID 4RLZ. The model is as shown in Fig. 1A, with all “hanging” bonds capped by protons. Blue vertical line indicates the geometry found in this PDB structure and blue circle represents the respective calculated energy.

In order to characterize this newly observed linear interaction between NH_3_^+^ and carbonyl group from an energetic standpoint we turned to density functional theory (DFT) calculations. A 2-molecule model system consisting of methylammonium ion and N-methylacetamide was constructed toward this goal (see Fig. 2H). The calculations were conducted using the program Q-Chem 4.3^5^ in conductor-like polarizable continuum water solvent C-PCM^6^ (see Methods for details). The relaxed potential energy scans have been performed over the parameter space (*r*, *θ*). The obtained two-dimensional energy map is shown in Fig. 2A; potential energy slices through the point corresponding to the geometry of interest, *r* = 2.7 Å, *θ* = 180°, are shown in Fig. 2B,C.

As can be seen from Fig. 2A, the linear geometry corresponds to a saddle point on the potential energy surface. The linear interaction (Fig. 2H) offers 2 kcal/mol stabilizing energy, whereas the hydrogen bond (Fig. 2G) offers ca. 5.5 kcal/mol. Figure 2C illustrates the scenario where the linear geometry is smoothly transformed into the hydrogen-bonded geometry while maintaining the overall planar arrangement. The same effect can be achieved by performing the rotation in an orthogonal direction; we have investigated this case separately and found that the dependence of energy on *θ* remains similar (see Fig. S3a). Other degrees of freedom also proved to be largely inconsequential. For instance, rotating N-methylacetamide about the pivot on O atom does not change the energy, see Fig. S3c,d. We conclude that the linear interaction NH_3_^+^ – carbonyl is essentially fully parameterized by the two coordinates, *r* and *θ*, and its energetics is nearly completely characterized by the data in Fig. 2A–C. Of note, standard hydrogen bond shows a significant dependence on the donor-acceptor distance as well as two characteristic angles^7^, i.e. has more sophisticated directionality properties than the newly described linear interaction.

According to Fig. 2C, the energy difference between the NH_3_^+^ – carbonyl hydrogen bond (stronger interaction) and NH_3_^+^ – carbonyl linear arrangement (weaker interaction) amounts to ca. 3.5 kcal/mol. On the other hand, comparing the populations of the two respective clusters in Fig. 1B points toward the free energy difference of 1.7 kcal/mol (if one assumes that PDB coordinates are representative of the structures at physiological temperature). Given that the data in Fig. 2C pertain to the simple model system, whereas the data in Fig. 1B are characteristic of the elaborate protein architectures, one should not expect a quantitative agreement between the above two estimates. In fact, it is rather satisfying that the two values are qualitatively consistent with each other.

Now recall that in the context of protein structure linear interaction NH_3_^+^ – carbonyl does not usually occur in isolation, but rather in combination with regular hydrogen bonds (see Fig. 1A). Specifically, among all examples of this linear interaction in the PDB-extracted set of high-quality protein structures, 61.4% of geometries feature three or more hydrogen bonds centered on the NH_3_^+^ group (this statistic includes bifurcated hydrogen bonds). Additionally, 20.5% of geometries have two hydrogen bonds. Apparently, the truly stable configuration involves NH_3_^+^ group engaged in three hydrogen bonds plus a linear interaction with an eligible carbonyl group, as illustrated in Fig. 1A. In this manner lysine ε-ammonium group fully utilizes its “bonding” potential. However, we may not always see all three hydrogen-bonded partners in the crystal structures – for example, if they include water molecules that have not been resolved in crystallographic model. Furthermore, in some cases lysine NH_3_^+^ groups form hydrogen bonds with symmetry-related molecules in the crystal lattice (not accounted for in our analyses). Alternatively, steric constraints encountered in the structure may prevent one or two hydrogen bonds from forming. While acknowledging such “incomplete” arrangements, we focus on the “complete” configuration such as shown in Fig. 1A.

To investigate such more extended system, we have constructed a 5-molecule model based on the coordinates 4RLZ, see Fig. 1A. This model includes one water and two formamide molecules, imitating the hydrogen-bonded partners of the lysine NH_3_^+^ group. Instead of N-methylacetamide, which has been previously used to imitate a peptide plane, we now use a smaller formamide molecule. This is necessary because the bulkier N-methylacetamide tends to pack against the hydrogen-bonded ligands, which to some degree obscures the effect of linear interaction. Using this PDB-extracted geometry as a starting point we have conducted the energy scans similar to the ones previously carried out for the 2-molecule model. In doing so, we have fixed the coordinates of the methylammonium ion and its three hydrogen-bonded ligands, while the formamide molecule responsible for the linear interaction has been moved by varying *r* and *θ*. Hence, we model hydrogen-bonded Lys side chain as a part of the static protein matrix – and treat its linear interaction with carbonyl as a weak perturbation.

The results of the energy calculations using 5-molecule model are summarized in Fig. 2D–F. Obviously, the energy surface representative of the linear interaction has changed from the saddle to the well, see Fig. 2D. This is understandable since the three hydrogen-bonding sites are now all occupied and the linear arrangement is the only energetically favorable arrangement that remains available to the fourth NH_3_^+^ ligand. Importantly, the “energy well” seen in Fig. 2D broadly corresponds to the cluster 2 in the PDB-based density map Fig. 1B.

It is worth noting that the favorable energetic effect of the linear interaction is similar to the one obtained for the 2-molecule model, cf. Figure 2B and Fig. 2E. In other words, adding three hydrogen bonds to the model has little effect on the energy of the linear interaction. More accurately, the energy of the linear interaction has been slightly attenuated, 1.8 vs. 2.0 kcal/mol. In principle, this is a reasonable outcome given that hydrogen bonds and linear interactions are mainly electrostatic in nature, see below, and therefore are expected to compete against each other (it is clear, however, that the small energy difference of 0.2 kcal/mol should not be overinterpreted considering the different makeup of the two models). We conclude that 2 kcal/mol is the sound estimate of the energy associated with the linear interaction, including the situation where NH_3_^+^ is hydrogen-bonded. Bear in mind, however, that this result does not directly reflect on the contribution of the linear interaction into protein stability. In order to evaluate such contribution, one would need to take into consideration lysine desolvation penalty, as well as potential transient contacts made by the NH_3_^+^ group in the disordered protein state – which is generally far from trivial^8,9^.

In essence, lysine NH_3_^+^ group, which makes three hydrogen bonds, can further improve its favorable binding energy by forming an axial interaction with a carbonyl group. This additional interaction is worth ca. one half of the hydrogen-bond energy. As such it is a meaningful complement to the lysine side-chain energy balance. The Protein Data Bank contains many examples of this “3 + 1” interaction scheme; the geometries found in the PDB are consistent with the energy minimum predicted in our model calculations (cf. blue vertical lines in Fig. 2E,F).

One important question concerns the nature of the linear interaction – is it mainly electrostatic, or does it have a partial covalent character, similar to hydrogen bonds? To address this question we have used the Natural Bond Orbital (NBO) analysis^10^. The calculations were conducted for geometry-optimized 2-molecule system methylammonium ion – formamide, and also for the formamide dimer that was employed as a model for the conventional hydrogen bond. As it turns out, linear interaction indeed has partially covalent character – mainly due to the donor-acceptor interactions involving lone electron pairs of the carbonyl oxygen and the antibonding orbitals associated with C^ε^-N^ζ^, N^ζ^-H^ζ1^, N^ζ^-H^ζ2^, and N^ζ^-H^ζ3^ bonds. However, the corresponding NBO stabilization energies are very small, an order of magnitude smaller than the corresponding values for the standard hydrogen bond (see Table S2 and Fig. S4). Therefore we conclude that the linear interaction has only minimal covalent character, but rather is dominated by electrostatics.

An interesting additional insight can be obtained from the analyses of NMR nuclear spin-spin couplings, which may exist despite the very weak covalency of the linear interaction^11,12^. Specifically, we are interested in “through-space” coupling between ^15^N^ζ^ and carbonyl ^13^C’. The DFT calculations using 2-molecule model predict the coupling constant of −1.4 Hz for the system in vacuum and −0.7 Hz for the system in aqueous PCM solvent. For the 5-molecule model based on x-ray coordinates both vacuum and PCM calculations predict the value −0.4 Hz. This is similar in magnitude to *J*-coupling constants across backbone hydrogen bonds that have been successfully measured for a number of small proteins^13,14^. It is well known, however, that NH_3_^+^ groups typically suffer from rapid solvent exchange, which makes them a difficult target for such measurements^15^.

**Figure 3:**
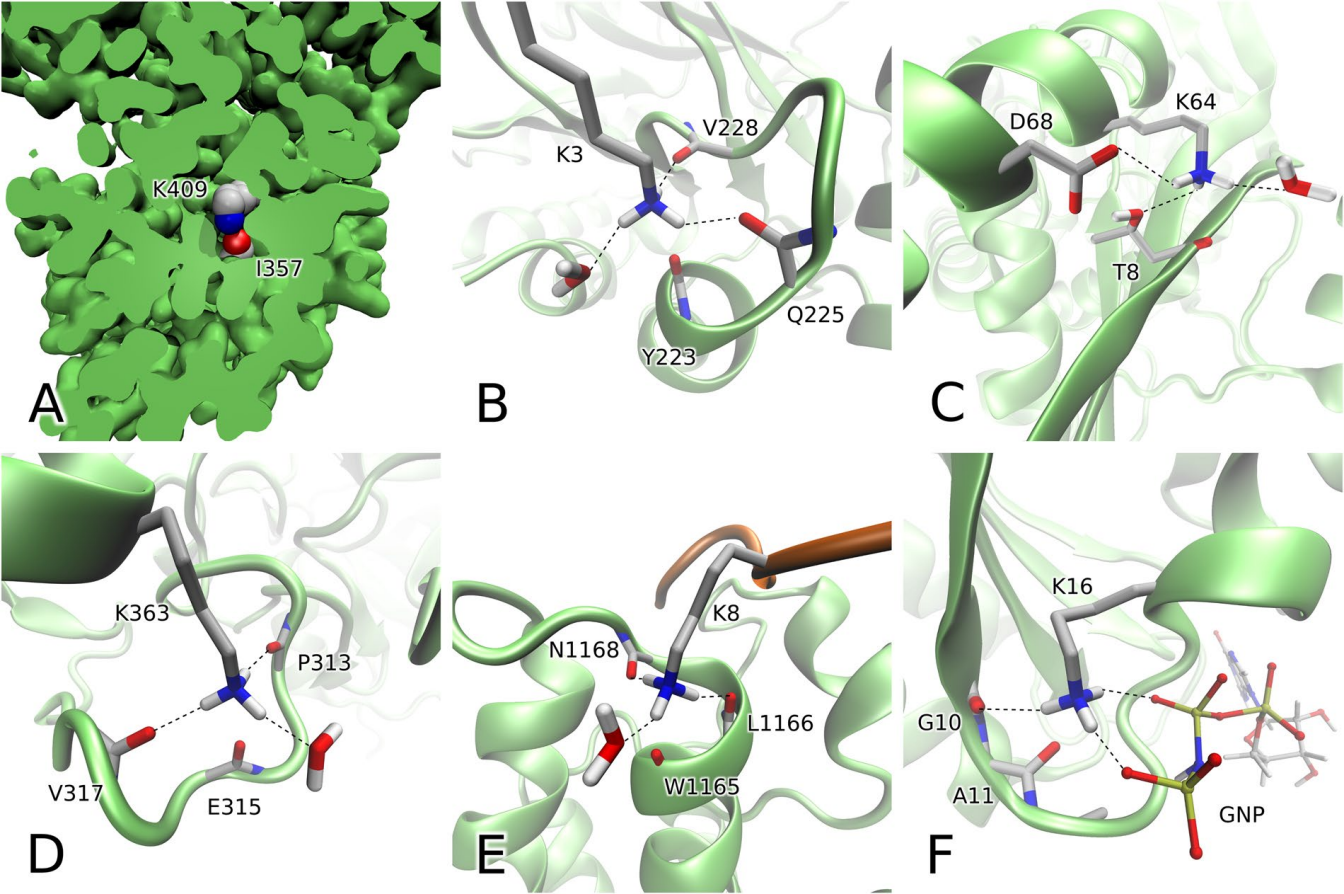
Selected examples of linear lysine-carbonyl interaction from the PDB-extracted set of high-resolution protein structures. (**A**) Linear interaction in the protein interior, PDB ID 4AC7^47^. (**B**) Linear interaction as an element of C-capping in α-helix, PDB ID 2J27^48^; the interaction involves carbonyl from the next-to-last residue in α-helix, Y223. (**C**) Linear interaction providing protection to the outer strand of the β-sheet, PDB ID 1NU5^49^. (**D**) Linear interaction in the turn region, PDB ID 4RLZ^45^. (**E**) Intermolecular linear interaction involving acetylated histone H4 peptide and bromodomain from transcription coactivator CBP, PDB ID 4N4F^21^, this arrangement can be described as “intermolecular helix capping”. (**F**) Linear interaction in the P-loop of human H-Ras p21 loaded with non-hydrolyzable analog of GTP, PDB ID 5P21^50^.

Since linear lysine-carbonyl interaction is a recurring motif in proteins, it is interesting to place this interaction in the context of protein structure. Typically, this interaction occurs near the protein surface, although we have also identified a number of exceptions where it is found in the protein core, see Fig. 3A. Addressing this latter situation, we can draw a parallel to buried salt bridges. In the low-dielectric-constant environment of protein interior, salt-bridging side chains form strong electrostatic interactions; however, the resulting favorable enthalpy is almost completely offset by a large desolvation penalty^16,17^. A similar compensation effect can be expected for linear lysine-carbonyl interactions that are sequestered in the protein core.

Parsing of the PDB-extracted dataset reveals that 89% of all linear interactions occur within one protein chain, while 11% are interchain contacts. Linear interactions can also occur in a form of crystal contacts between symmetry-related molecules in the crystal lattice. Considering secondary-structure preferences, lysine residues involved in linear interaction are no different from the general population of lysines, i.e. show no special preferences. On the other hand, their counterpart carbonyl groups do have distinctive conformational preferences: they strongly disfavor β-sheet (as expressed by a factor 0.26) and less so α-helices (0.61), while showing moderate preference for coil (1.27) and more substantial preference for turns (2.06).

The case of α-helices deserves a special discussion. Starting from N-terminus of an α-helix, all carbonyl groups are engaged in canonical CO ··· HN hydrogen bonds – except for three carbonyls at the C-terminal end of the helix which lack HN counterparts in a standard helical arrangement^18^. Although technically possible, lysine side chain rarely forms linear interaction with a hydrogen-bonded carbonyl in the N-terminal portion of the helix. On the other hand, lysine side chains often connect to one of the eligible carbonyls at the C-terminal end of α-helices. As it turns out, the last residue in α-helix is a favored site to form linear interaction with the corresponding propensity 1.9, while next-to-last residue has even stronger preference, 3.6. This leads us to suggest that linear interactions have a role in C-terminal helix capping. Similar observations can be made for 3_10_ helices (see Fig. S6 for additional information). In relative terms, helix-capping motifs involving linear NH_3_^+^ – carbonyl interactions are rare. In our PDB-extracted dataset only 0.4% of all α-helices are capped in this manner (note that capping motifs involving side chains are generally infrequent in the C-termini of α-helices^19^). However, on the absolute scale this translates into 2,122 unique examples of linear interaction occurring as a part of the C-terminal helix cap (see Fig. 3B for illustration). Evidently, in each of these instances linear interaction can have a role in stabilizing the C-terminal end of the helix and thereby could exert influence on protein stability and function.

Not too many linear interactions originate from carbonyl groups in β-strands. For those carbonyls that are engaged in canonical CO ··· HN hydrogen bonds, linear interactions are possible, but exceedingly rare. In contrast, those carbonyls that are located in the outer strands and are oriented outward are more likely to form linear interactions. There are examples of linear interaction protecting the outer strand of β-sheet (see Fig. 3C) whereby the presence of charged ε-ammonium group over the outer edge of the sheet prevents it from connecting with a β-sheet from another protein molecule and thus avoids unwanted dimerization^20^. In a number of cases linear interaction involves free carbonyl groups of the last (C-terminal) residue in β-strand, apparently providing stabilization akin to the helix capping and reducing terminal fraying in the β-sheet.

Finally, it is not surprising that linear interactions are often encountered in turns. Indeed, turns provide an “open” topology, where several hydrogen-bond acceptors can converge on one point to engage lysine side-chain NH_3_^+^ group. In addition, a free carbonyl is frequently available to form an axial interaction and thus complete the arrangement (illustrated in Fig. 3D).

Although relatively rare, linear interactions are expected to be of consequence in many specific proteins or protein complexes. For example, illustrated in Fig. 3E is the linear interaction at the interface between doubly-acetylated histone H4 tail and bromodomain from transcription coactivator CBP^21^. In this case, the H4 peptide contributes lysine side chain, which is accommodated by the C-terminal turn of helix B from the bromodomain, creating a full complement of interactions for lysine ε-ammonium group, i.e. three hydrogen bonds (two toward backbone carbonyls and one toward the ordered water molecule) plus linear interaction with the carbonyl in −2 position from the C-terminal end of the helix. Of note, the binding of H4 to bromodomains is typically transient, with affinity in the range from tens to hundreds of micromoles^22^. In this situation, the linear interaction shown in Fig. 3E is expected to provide a meaningful contribution to the binding affinity. Indeed, it has been observed that the lysine residue at hand is one of the two key binding residues^21^. The recognition of acetylated H4 by bromodomains activates gene transcription; this mechanism has been identified as a potential target for epigenetic cancer therapy, leading to intense search for competitive inhibitors^23,24^.

An even more interesting example of linear interaction is found in the structure of the well-known oncoprotein H-Ras p21. This protein belongs to the ubiquitous P-loop NTPase fold and therefore contains the signature P-loop sequence GxxxxGK(S/T). The conserved lysine residue in this motif is particularly important for NTP loading and hydrolysis: its ε-ammonium group forms hydrogen bonds with γ- and β-phosphates, as well as carbonyl of the conserved glycine residue at the beginning of the P-loop. As it turns out, in the Ras proteins the consensus lysine K16 can additionally establish linear interaction with the carbonyl from the second residue in the P-loop, A11. This arrangement, illustrated in Fig. 3F, features a pair of consecutive residues, where conserved glycine G10 forms hydrogen bond with the lysine NH_3_^+^ group and the next residue A11 forms linear interaction with the same group. Of note, the hydrogen bond is shifted slightly away from its standard orientation (C^ε^-N^ζ^-O angle 93°), making additional room for the linear interaction (C^ε^-N^ζ^-O angle 151°).

In the Protein Data Bank we have identified 159 structures of Ras and Ras-like proteins featuring linear interaction between the conserved lysine in position 7 and residue in position 2 within the P-loop (in other Ras-family structures the interaction falls just outside the boundaries of the cluster 2, Fig. 1B). Furthermore, we found multiple structures of other P-loop NTPases that feature the same characteristic linear interactions: adenylate kinases, e.g. 1ZIN^25^, mitochondrial ATP synthases, e.g. 2JDI^26^, elongation factors, e.g. 4LBW^27^, myosin and kinesin motor domains, e.g. 1LVK^28^ and 2ZFI^29^, etc. The P-loop NTPase fold is by far the most common protein fold in eukaryotes; many of its functions, such as genome replication and translation, are indispensable for life^30^. Evidently, the linear interaction discussed above contributes toward stabilization of the P-loop in a requisite conformation, which is highly important for the catalytic activity of NTPase^31^.

One may wonder if linear interaction occurs only among NH_3_^+^ – carbonyl pairs in proteins, or similar motifs may also arise in other contexts. The analysis of the PDB-derived dataset showed that oxygen atom of crystallographic water is also often found near the symmetry axis of the ε-ammonium group. Such water oxygens form a distinct cluster on the density map akin to the cluster 2 in Fig. 1B, but centered somewhat farther away from N^ζ^ atom (see Fig. S7a). Furthermore, it turns out that hydroxyl oxygen atoms from Ser, Thr and Tyr side chains are also frequently localized in the area of cluster 2 (see Fig. S7b). These examples suggest that sp3 oxygen can interact with the NH_3_^+^ group in a similar manner to the sp2 oxygen; the details of this former interaction await future investigation.

In addition, we have also identified several examples of linear arrangement involving lysine NH_3_^+^ group and various sites in nucleic acids, e.g. carbonyl of the nitrogenous base (PDB ID 1C9S^32^) or ribose hydroxyl (PDB ID 1SDS^33^). However, there is no adequate statistics for such pairs, which makes it difficult to decide whether these examples represent a reproducible structural motif.

In this connection it is also interesing to note that carboxylic oxygens belonging to the Asp and Glu side chains do not produce a clear-cut cluster that can be associated with linear interaction (see Fig. S7c). It appears that COO^–^ can usually force its way into a more favorable hydrogen-bonded position by displacing a weaker acceptor, such as carbonyl. As a result, COO^−^ ··· NH_3_^+^ salt bridges almost invariably occur in a form of hydrogen bonds, while linear arrangement for this ion pair is rare and does not manifest itself as a distinct structural motif (a more nuanced discussion would require the knowledge of carboxyl protonation status).

In conclusion, lysine NH_3_^+^ – carbonyl linear interaction provides an interesting example of the previously unidentified structural motif. It can successfully complement hydrogen bonds, providing a meaningful addition to the stabilization energy. This motif finds some distinctive uses in protein architecture, e.g. in helix capping; it also turns out to be an integral part of important protein sites, such as the P-loop in nucleoside triphosphate hydrolases. Other examples of apparent linear interaction between NH_3_^+^ and various polar moieties also exist and warrant further investigation.

## Methods

### PDB analyses

A subset of high-quality protein x-ray structures has been extracted from Protein Data Bank on 06.09.2017 using the following criteria: resolution is 2.0 Å or better and *R*_*free*_ is 0.25 or better. Further analysis was restricted to the first model from each PDB file. All atoms with alternate conformations have been ignored. The program HBPLUS^34^ has been used to identify hydrogen bonds and the program STRIDE^35^ has been used for secondary structure classification. We have also used other selection criteria: (*i*) resolution is 1.5 Å or better, *R*_*free*_ is 0.20 or better and/or (*ii*) sequence identity between any two structures in the subset is 90% or lower. The results from these additional analyses are presented in Fig. S1. The propensity of linear interactions toward certain elements of secondary structure is calculated as explained in the caption of Fig. S6.

### Quantum chemistry calculations

All calculations were performed using Q-Chem (unless explicitly stated, see below). To calculate the energies of 2-molecule model system methylammonium ion – N-methylacetamide, the following procedure has been adopted. The initial geometry has been generated as illustrated in Fig. 2G,H for a given setting of *r* and *θ*. For each of the three methyls as well as NH_3_^+^ group we generated two different rotamers differing by 60°, resulting in a total of 16 different starting geometries. Each of these geometries has been optimized using B3LYP functional^36,37^ with 6-31G(d) basis set^38,39^ while keeping the coordinates of C^ε^, N^ζ^ and O atoms fixed. The resulting refined models were used to conduct single-point energy calculations employing ωB97X-D functional^40^ with cc-pVQZ basis set^41^. The lowest energy (among the 16 models) was chosen for plotting in Fig. 2A–C. This tactic has allowed for consistently good optimization of proton coordinates. To model a polar environment on the protein surface, all calculations were carried out using conductor-like C-PCM solvent^6^.

Similar protocol has been used to calculate the energies in 5-molecule model system, Fig. 2D–F. In this case, the coordinates of all atoms belonging to the hydrogen-bonded ligands of NH_3_^+^ have been fixed according to the crystal structure 4RLZ. The N-methylacetamide molecule has been replaced in this model with the smaller formamide molecule; the effect of this replacement on the calculated energy is modest (cf. Table S1). *J*-coupling constants have been computed for optimized geometries at the same level of DFT as energies using the Mixed option^42^ in the Gaussian program^43^. NBO (Natural Bond Orbital) analysis was performed on geometry-optimized model using NBO 5.0 program^44^ (available as a part of Q-Chem package) at B3LYP/6–31G(d) level.

### Data availability statement

All data are available from the authors upon request.

## Electronic supplementary material

Supplementary Information
