# Supplementary material for: A new structural arrangement in proteins involving lysine NH3+ group and carbonyl
Source: Sci Rep. 2017 Nov 27;7:16402. doi: 10.1038/s41598-017-16584-y (PMC5704018; doi:10.1038/s41598-017-16584-y)
Supplement: Supplementary file 1 — Supplementary Information [file 41598_2017_16584_MOESM1_ESM.pdf]

# Supplementary Information

## A new structural arrangement in proteins involving lysine $NH_3^+$ group and carbonyl.

Olga N. Rogacheva,[1] Sergei A. Izmailov,[1] Lyudmila V. Slipchenko,[2] and Nikolai R. Skrynnikov[1,2*]

[1] Laboratory of Biomolecular NMR, St. Petersburg State University, St. Petersburg 199034, Russia

[2] Department of Chemistry, Purdue University, West Lafayette IN 47907, USA

* Corresponding author. E-mail: nikolai@purdue.edu

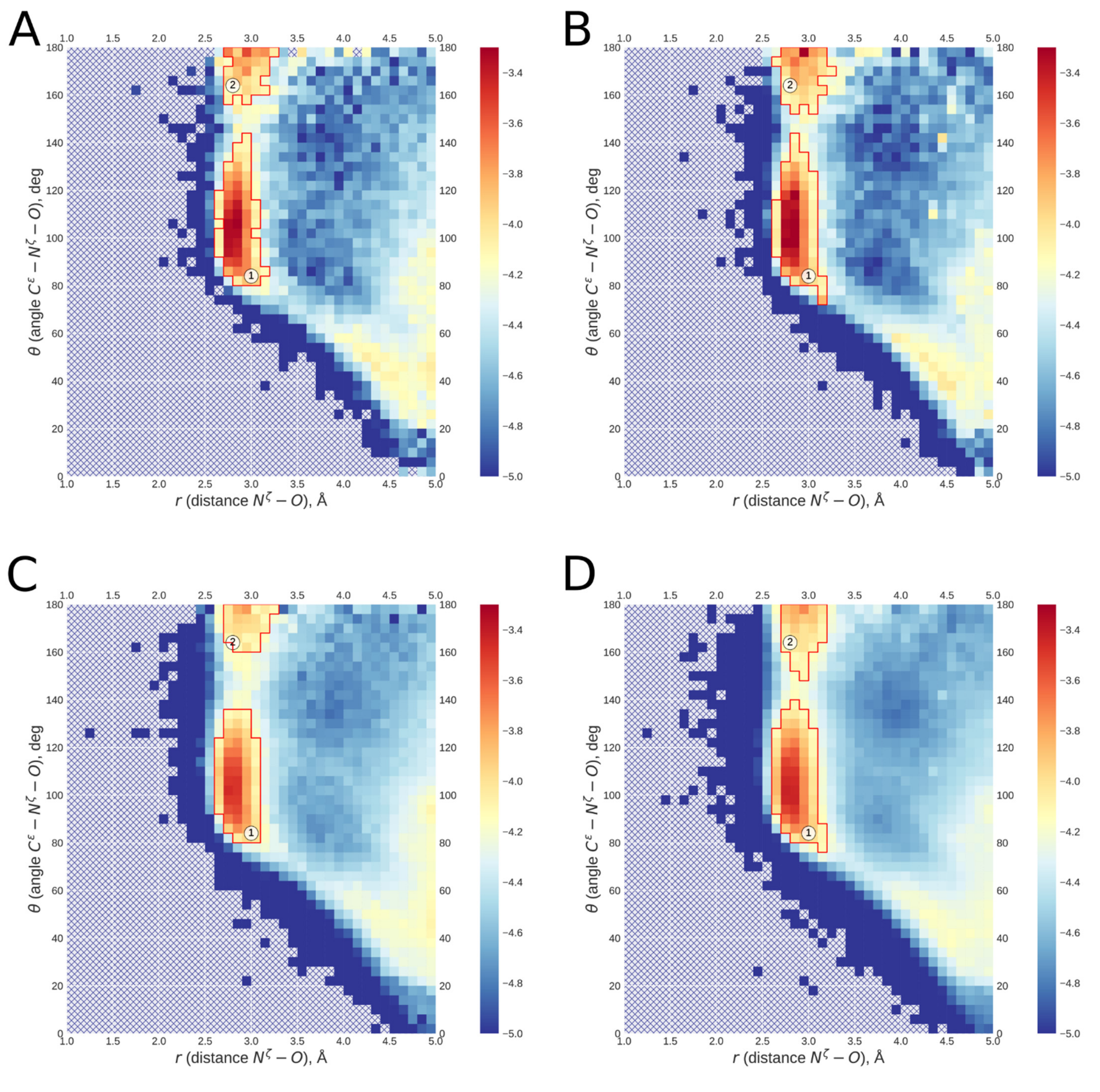


Figure S1. Maps illustrating relative positioning of lysine $NH_3^+$ and proximal carbonyl groups based on analysis of selected x-ray structures in the Protein Data Bank. The structures have been selected using the following criteria: (**A**) resolution 1.5 Å or better, $R_{free} = 0.2$ or better, sequence identity between any two structures in the subset 90% or lower (3,609 selected structures); (**B**) resolution 1.5 Å or better, $R_{free} = 0.2$ or better (8,632 selected structures); (**C**) resolution 2.0 Å or better, $R_{free} = 0.25$ or better, sequence identity between any two structures in the subset 90% or lower (17,597 selected structures); (**D**) resolution 2.0 Å or better, $R_{free} = 0.25$ or better (47,388 selected structures, same as in Fig. 1b).

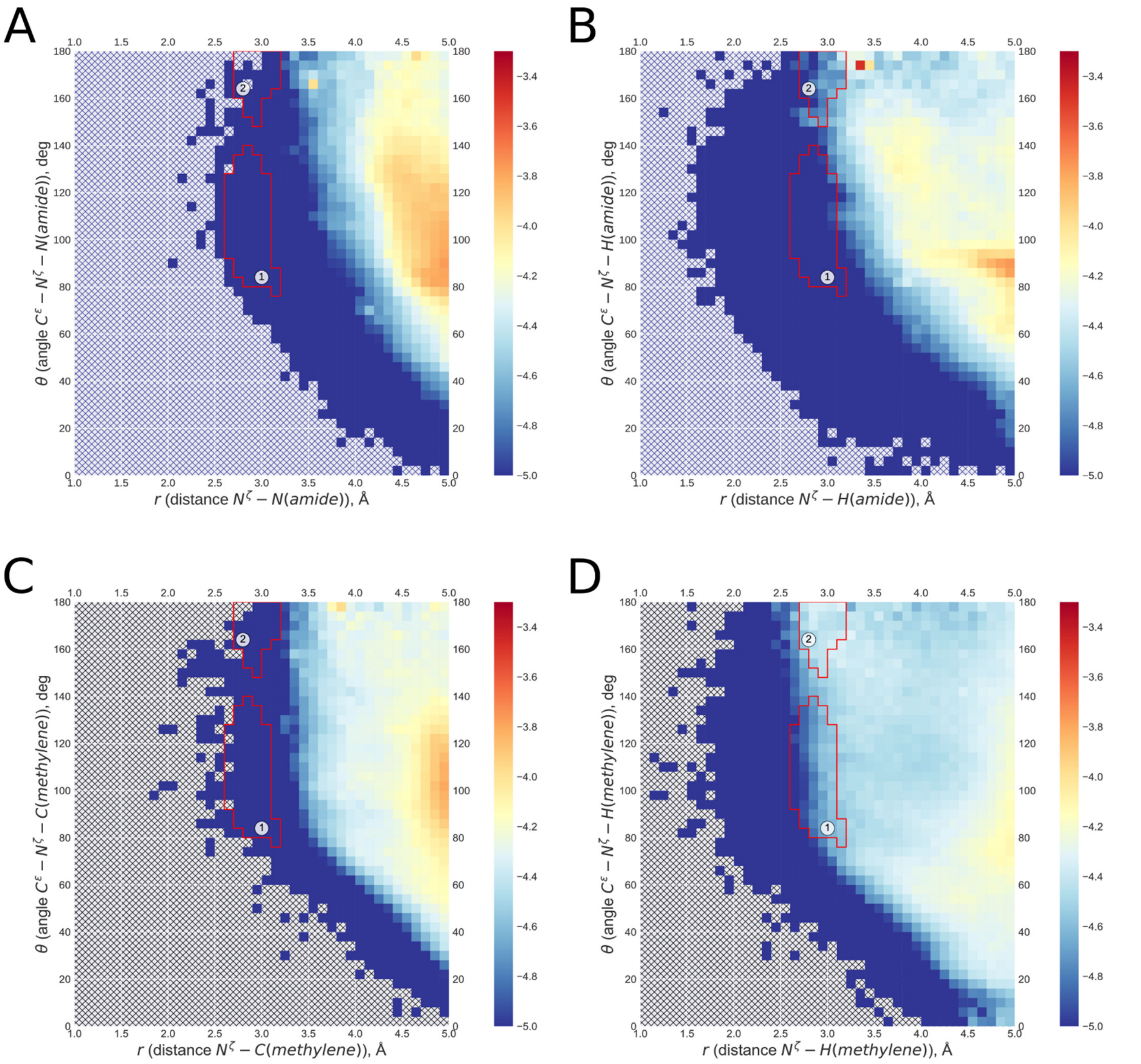


Figure S2. Maps illustrating relative positioning of lysine $NH_3^+$ and proximal (**A**) backbone N atoms, (**B**) backbone $H^N$ atoms, (**C**) side-chain methylene C atoms, and (**D**) side-chain methylene H atoms. The data are extracted from the same set of x-ray structures as in Fig. 1b. The contours 1 and 2 are reproduced from Fig. 1b. Clearly, both of these regions do not constitute distinct clusters for the data at hand. In preparing the plots (C, D) we have ignored those methylene groups that belong to the same lysine side chain as the analyzed $NH_3^+$ group. The outlier at $r = 3.3$ Å, θ = 172° in the panel (B) arises from multiple structures of endothiapepsin.

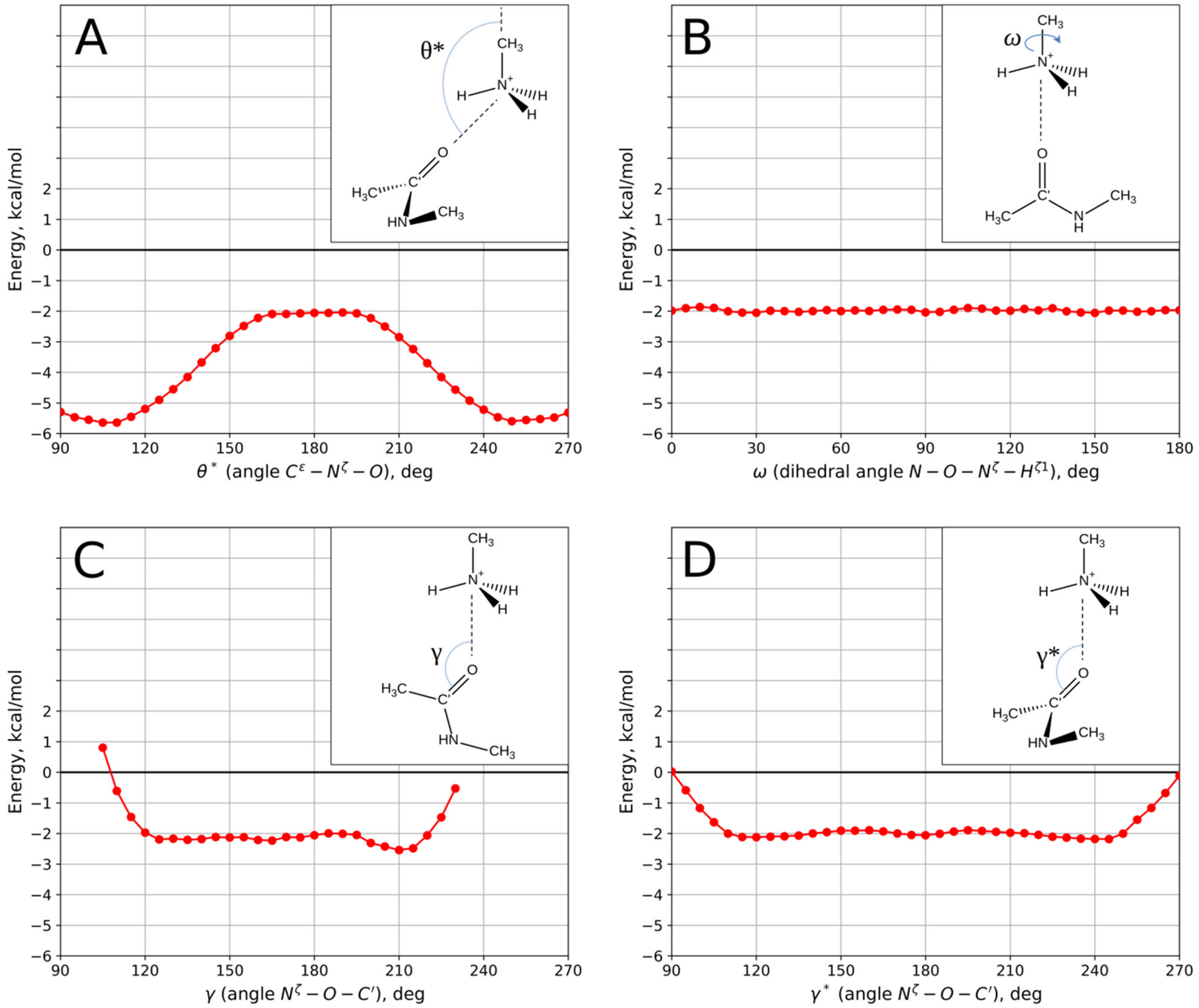


Figure S3. DFT energy scans for 2-molecule model (methylammonium ion and N-methylacetamide) representative of the linear interaction between lysine $NH_3^+$ group and carbonyl. This figure is complementary to Fig. 2 and demonstrates that the energy of linear interaction essentially does not depend on structural parameters other than $\theta$ and $r$. The definitions of the variables of interest, $\theta^*$, $\omega$, $\gamma$ and $\gamma^*$, are illustrated in the insets. The angle $\theta^*$ is formally equivalent to $\theta$ (the difference lies in the orientation of the NMA peptide plane); likewise, the angle $\gamma^*$ is formally equivalent to $\gamma$. Prior to energy calculations, model geometries have been optimized while maintaining fixed coordinates for the atoms $C^{\varepsilon}$, $N^{\zeta}$ and O; for calculations in panels (C) and (D), we have additionally fixed the coordinates of the atom C'. In this connection note that positions of $NH_3^+$ protons are shown for illustrative purpose only – in fact, proton coordinates have been independently optimized for each individual model. Other details of energy calculations are the same as described in the main text.

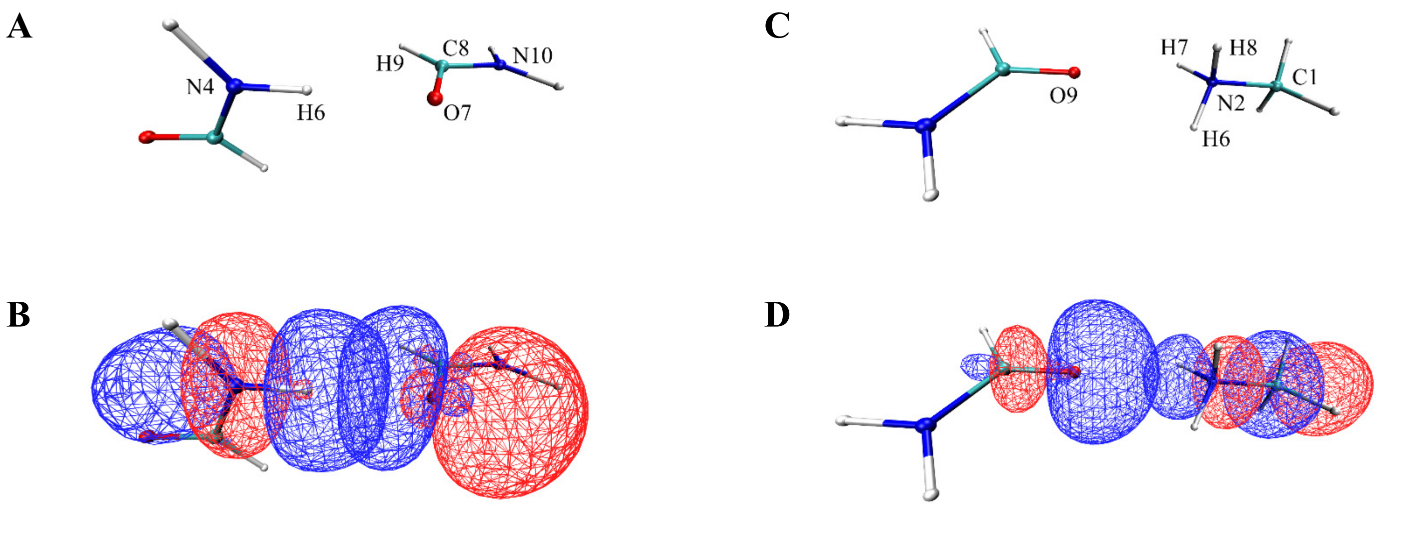


Figure S4. Models representing (**A**,**B**) hydrogen bond between two formamide molecules and (**C**,**D**) linear interaction between methylammonium ion and formamide. Shown are the pairs of donor/acceptor NBO orbitals that make the largest contribution to the stabilizing energy $\Delta E^{(2)}$ (corresponding to the bold entries in Tab. S2). The red- and blue-colored isosurfaces have negative and positive isovalues (±0.03), respectively. The geometries have been optimized in the PCM solvent as described in the legend of Tab. S1.

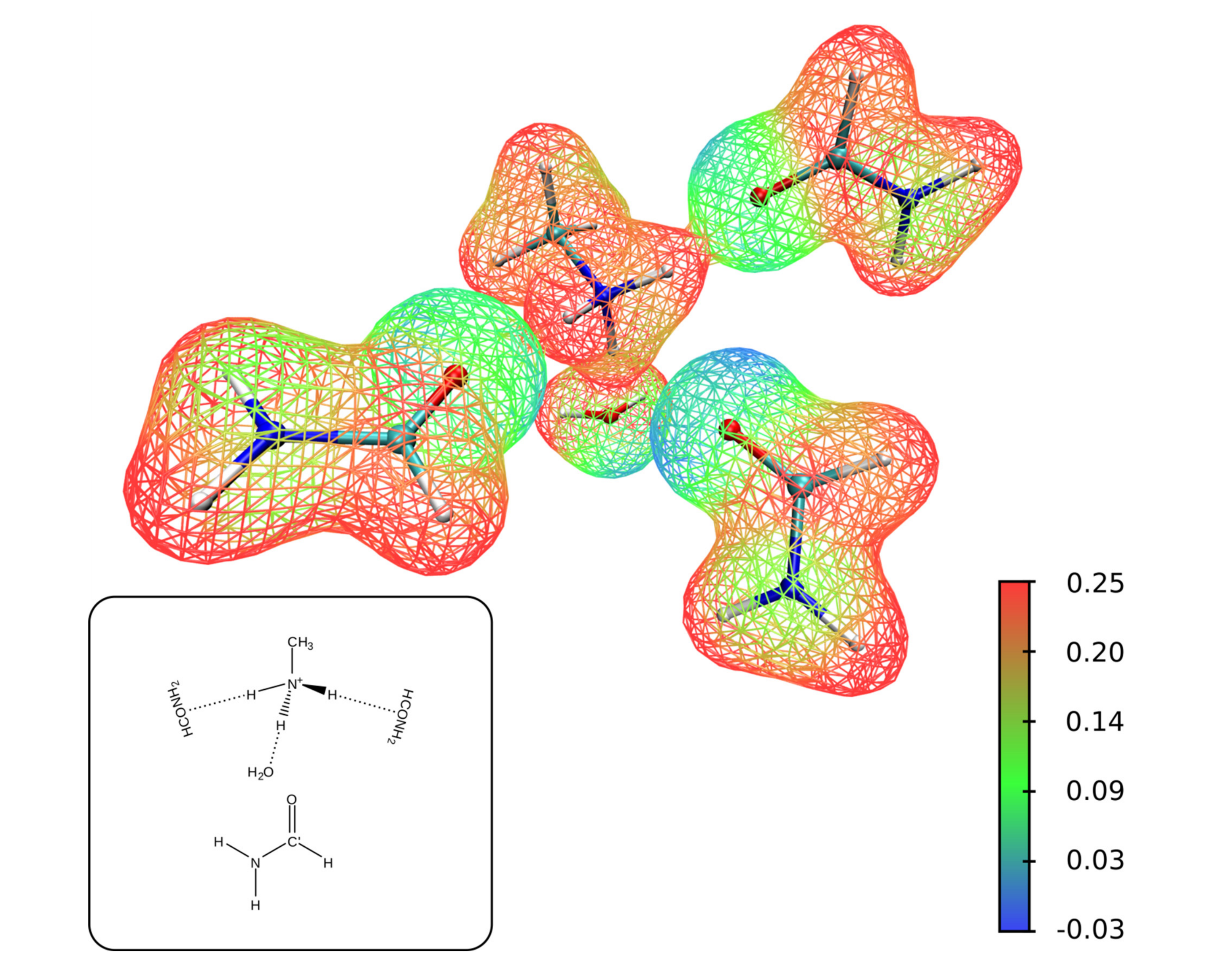


Figure S5. Electron isodensity surface at 0.025 *e*/bohr$^3$ calculated for the 5-molecule model derived from PDB ID 4RLZ (see Fig. 1a; additionally illustrated in the inset). The surface is colored according to the electrostatic potential with the scale as indicated on the side (units of hartree/*e*). The calculations have been conducted at the ωB97x-D/cc-pVQZ level of theory, same as described in the main text.

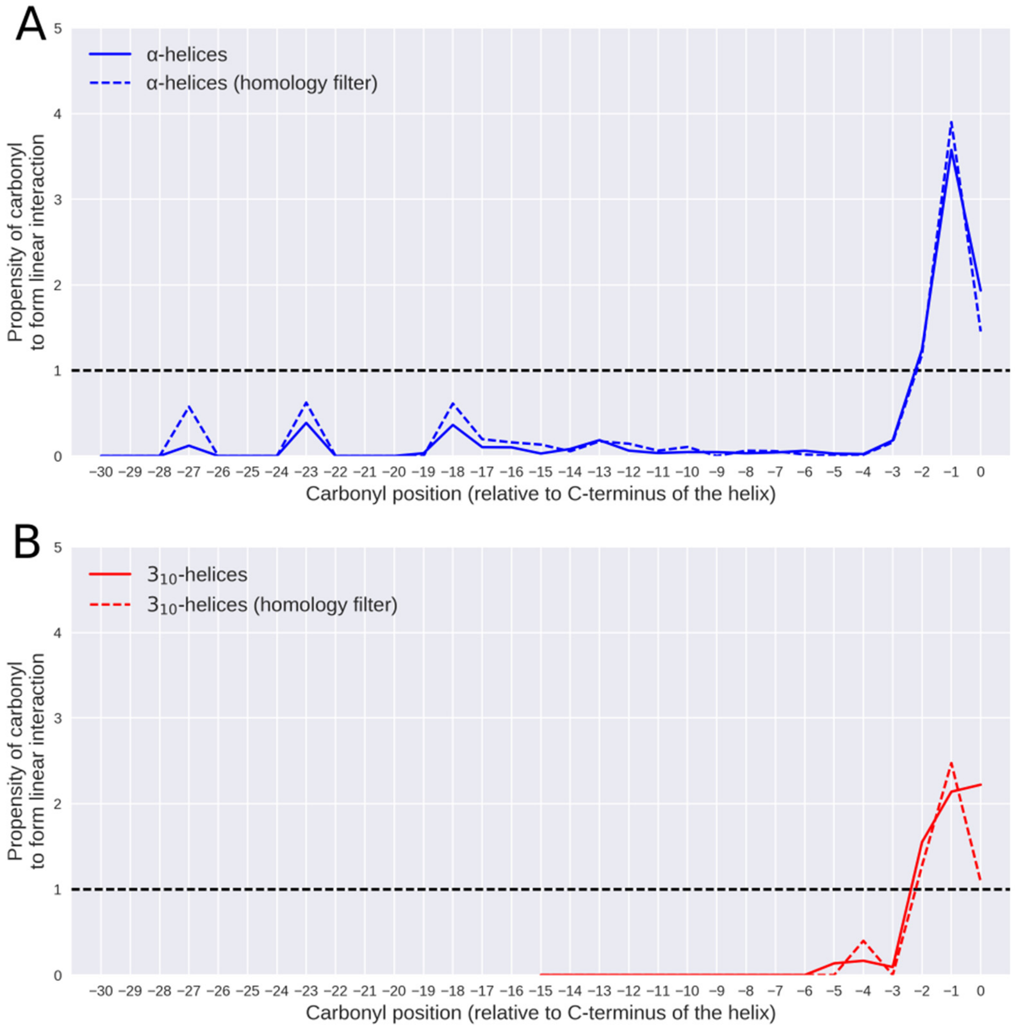


Figure S6. Propensity of backbone carbonyl sites in (**A**) α-helices and (**B**) $3_{10}$-helices to form linear interaction with lysine $NH_3^+$ group. The results from the main PDB-extracted dataset (47,388 structures) are shown with continuous lines. For instance, to quantify the propensity of the last (C-terminal) carbonyl site in an α-helix to form linear interaction we use the formula $(n_{lin}^0 / N_{lin}) / (n_0 / N)$, where $N_{lin}$ = 11,719 is the number of examples of linear interaction in our collection (i.e. number of entries in cluster 2 in Fig. 1b), $n_{lin}^0$ = 608 is the number of linear interactions formed by carbonyls that occupy the last position in an α-helix, $N$ = 22,180,201 is the number of carbonyls in our PDB-extracted dataset, and $n_0$ = 595,243 is the number of carbonyls that occupy the last position in an α-helix. Using this convention, the propensities that are less than 1.0 signify negative preference and the propensities that are greater than 1.0 signify positive preference toward linear interaction. The tendency of linear interactions to cap α-helices in three C-terminal positions (0, -1 and -2) is a notable feature of this graph (see main text for discussion). Aside from these three positions, linear interactions rarely originate on those carbonyl sites that are involved in canonical CO···NH hydrogen bonds. Similarly, linear interactions often cap $3_{10}$-helices in two C-terminal positions (0 and -1). In addition, there are many instances of linear interactions involving carbonyl in position -2, which does form CO···NH bond within the $3_{10}$-helix. In this connection one should bear in mind that short $3_{10}$-helices are often significantly bent,[1] which apparently creates geometry suitable for linear interaction. The results from the reduced PDB dataset containing only unique structures (17,597 structures) are shown with dashed lines. The significant difference between the continuous and dashed profiles for position 0 in $3_{10}$-helices is due to 230 crystallographic structures of carbonic anhydrase featuring this particular arrangement.

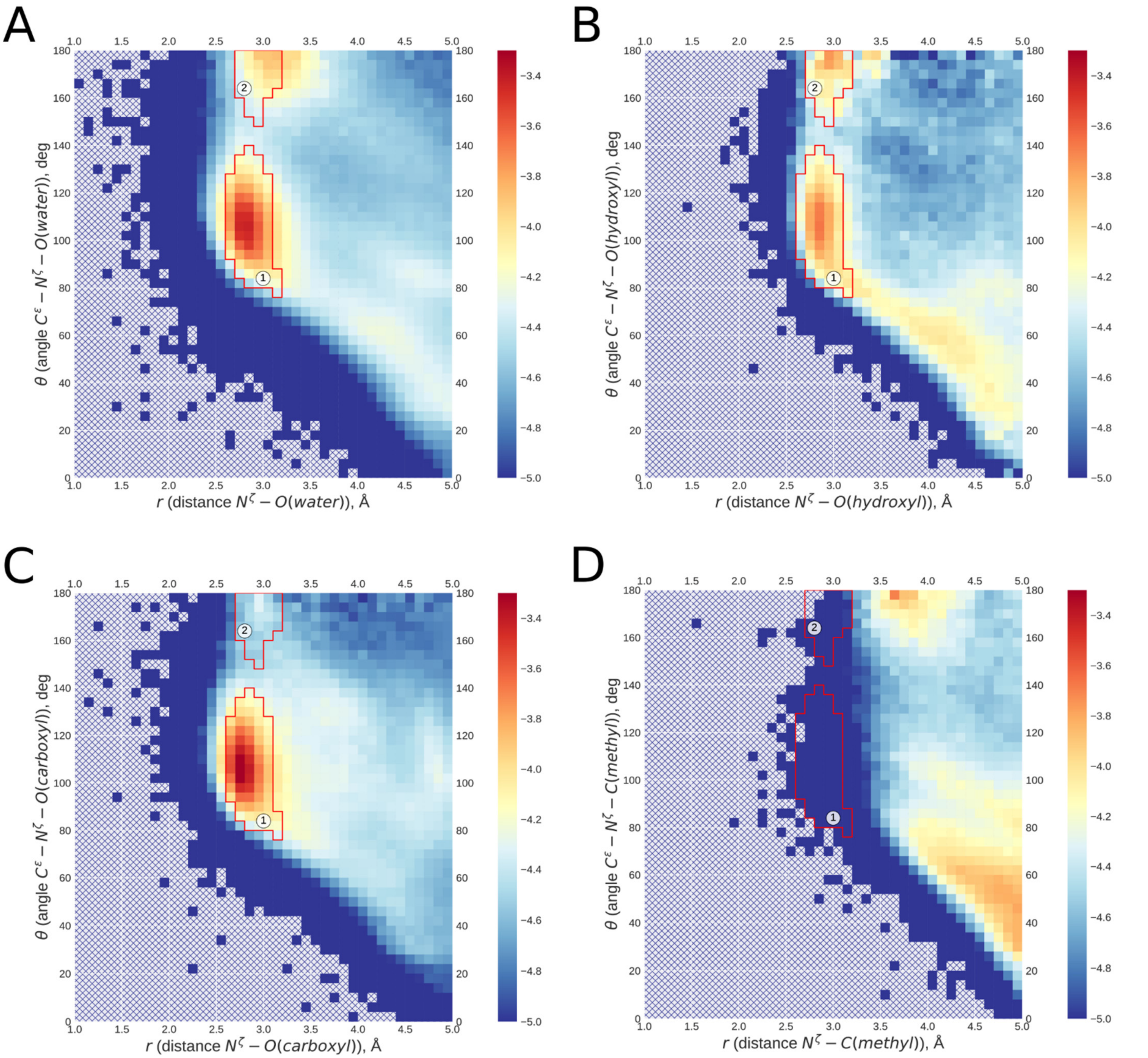


Figure S7. Maps illustrating relative positioning of lysine $NH_3^+$ and various proximal atoms: (**A**) oxygen from crystallographic water, (**B**) oxygen from side-chain hydroxyl groups, (**C**) oxygen from side-chain carboxylic groups and (**D**) carbon from side-chain methyl groups. The data are extracted from the same set of x-ray structures as in Fig. 1b; the contours 1 and 2 are reproduced from Fig. 1b. Of interest, methyls show certain tendency to pack against $NH_3^+$ groups in a form of a linear arrangement, with the corresponding cluster visible in the map at $r$ = 3.7 Å, $\theta$ = 180°, see panel (D). Our DFT and force-field calculations have shown that this is due to van der Waals packing that produces favorable stabilizing energy of ca. 0.5-1.0 kcal/mol. Indeed, it is the only comfortable spot for the methyl group in the situation when $NH_3^+$ is encircled by the three hydrogen-bond acceptors. The results illustrated in the other panels, (A-C), are discussed in the main text.

| | Hydrogen bond FA – FA | | Linear interaction FA – MA | | Linear interaction NMA – MA | |
|---|---|---|---|---|---|---|
| | gas | water (PCM) | gas | water (PCM) | gas | water (PCM) |
| distance N-O, Å | 2.9 | 2.9 | 2.6 | 2.8 | 2.5 | 2.7 |
| angle O-H-N, deg | 165 | 177 | 91 | 91 | 90 | 90 |
| angle C-O-N, deg | 104 | 112 | 179 | 179 | 180 | 180 |
| E (SPE), kcal/mol<br>including [*deformation correction*][a], kcal/mol | -7.2<br>[*0.5*] | -3.8[g] | -23.3<br>[*1.2*] | -1.6 | -26.4<br>[*1.6*] | -2.1 |
| E (CCSD(T)//CBS(3,4))[b], kcal/mol | -7.3 | | -24.4 | | | |
| E (EDA)[c], kcal/mol<br>including [*BSSE correction*][d], kcal/mol | -7.2<br>[*0.4*] | | -24.2<br>[*0.3*] | | -27.8<br>[*0.3*] | |
| Frozen density contribution, kcal/mol (%) | -3.3 (46%) | | -17.5 (72%) | | -18.8 (67%) | |
| Polarization contribution, kcal/mol (%) | -1.7 (24%) | | -5.5 (23%) | | -7.2 (26%) | |
| Charge-transfer contribution, kcal/mol (%) | -2.2 (30%)[f] | | -1.2 (5%) | | -1.8 (6%) | |
| E (SPE), kcal/mol<br>corrected for *BSSE* and *deformation* error | -6.8[f] | | -23.0 | | -26.1 | |
| E (EDA), kcal/mol<br>corrected for *BSSE* and *deformation* error | -6.7[f] | | -23.0 | | -26.2 | |
| "Through-space" scalar coupling $J(^{15}N,^{13}C)$[e], Hz | 0.2 | 0.2 | -1.0 | -0.5 | -1.4 | -0.7 |

Table S1. Calculated geometry and energetics of the simple 2-molecule models representative of backbone CO···HN hydrogen bond and linear $NH_3^+$ – carbonyl interaction. In the case of hydrogen bond model, constrained geometry optimizations at the B3LYP/6-31G(d) level of the theory were performed, whereby the distance N – O was varied with the step 0.1 Å and all other coordinates were fully optimized; the resulting models were ranked according to ωB97x-D/cc-pVQZ energies (SPE) to identify the best geometry. Similarly, in the case of linear interaction models, the angle $C^{\varepsilon} – N^{\zeta} – O$ was fixed at 180°, the distance N – O was varied on a grid with the step 0.1 Å and all other coordinates were optimized at each point on the grid (including deep optimization of methyl and $NH_3^+$ protons, see main text); the best geometry was selected based on ωB97x-D/cc-pVQZ energies. Abbreviations: FA – formamide, MA – methylammonium ion, NMA – N-methylacetamide, PCM – polarizable continuum model, SPE – single-point energy, EDA – energy decomposition analysis, BSSE – basis set superposition error, CCSD(T) – coupled cluster with single, double and perturbative triple substitutions, CBS – complete basis set.

a. Deformation energy has been calculated using a separate monomer/dimer SPE calculation.

b. Hartree-Fock energies were calculated with aug-cc-pVTZ and aug-cc-pVQZ basis sets by making use of the two-point extrapolation scheme described by Halkier *et al.*[2] to address basis convergence (using the recommended value α=1.63). Correlation energies were calculated using MP2 method with aug-cc-pVTZ and aug-cc-pVQZ basis sets in conjunction with another two-point extrapolation scheme due to Halkier *et al.*[3] Additionally, ΔCCSD(T) correction for the correlation energy has been calculated at aug-cc-pVDZ level.[4] The results of these calculations are similar to the EDA results in that they are unaffected by BSSE, but suffer from the deformation error.

c. Energy decomposition analysis using absolutely-localized molecular orbitals (ALMO EDA)[5] was performed on optimized geometries at the same level of theory (ωB97x-D/cc-pVQZ) as energy calculations described in the main text.

d. BSSE correction has been calculated in a form of counterpoise correction as implemented by Khaliullin *et al.*[5] For FA-MA and NMA-MA models representing linear interaction in vacuum, BSSE corrections proves to be much smaller than deformation corrections. This leads us to suggest that SPE calculations in PCM solvent, as described in the main text, are only minimally compromised by BSSE.

e. This includes scalar coupling constants through hydrogen bond, $^{3h}J(^{15}N,^{13}C')$, and through linear interaction, $^{2l}J(^{15}N^{\zeta},^{13}C')$. These constants are strongly dependent on the details of model geometry, which are somewhat different between the vacuum- and PCM-optimized models.

f. The results for hydrogen bond energy in vacuum are in agreement with those reported in the literature, see e.g. Morozov *et al.*[6]

g. The results for hydrogen bond energy in polar environment are consistent with the experimental evidence[7] (keeping in mind that the calculations have been conducted on an isolated fully optimized small-molecule system).

| FA-FA hydrogen bond | | | FA-MA linear interaction | | |
|---|---|---|---|---|---|
| Donor NBO | Acceptor NBO | Δ$E^{(2)}$, kcal/mol | Donor NBO | Acceptor NBO | Δ$E^{(2)}$, kcal/mol |
| BD(O7-C8) | BD*(N4-H6) | 0.19 | BD(C1-N2) | RY*(O9) | 0.25 |
| CR(O7) | BD*(N4-H6) | 0.22 | **$LP_1$(O9)** | **BD*(C1-N2)** | **0.50** |
| $LP_2$(O7) | RY*(H6) | 0.17 | $LP_1$(O9) | BD*(N2-H6) | 0.19 |
| $LP_1$(O7) | BD*(N4-H6) | 4.83 | $LP_1$(O9) | BD*(N2-H7) | 0.19 |
| **$LP_2$(O7)** | **BD*(N4-H6)** | **13.04** | $LP_1$(O9) | BD*(N2-H8) | 0.18 |
| | | | $LP_2$(O9) | BD*(N2-H6) | 0.15 |

Table S2. Stabilization energies Δ$E^{(2)}$ due to donor NBO – acceptor NBO interactions in the simple 2-molecule models representative of backbone CO···HN hydrogen bond and linear $NH_3^+$ – carbonyl interaction. The geometries have been optimized in the PCM solvent as described in the legend of Tab. S1. Abbreviations: FA – formamide, MA – methylammonium ion, CR – 1-center core pair, BD – 2-center bonding orbital, LP – 1-center valence lone pair, RY* – 1-center Rydberg orbital, BD* - 2-center antibonding orbital. The numbering of the atoms is shown in Fig. S4. Only those Δ$E^{(2)}$ contributions that exceed the threshold of 0.15 kcal/mol are included. The dominant contributions into Δ$E^{(2)}$ that are primarily associated with the hydrogen bond and linear interaction are typeset in bold and visualized in Fig. S4. Note that Δ$E^{(2)}$ (second-order perturbation theory energy shifts involving off-diagonal elements of the Kohn-Sham matrix[8]) are used by us only for the purpose of *comparative* analysis of the two interactions.

## Supplementary References


1. Barlow, D. J. & Thornton, J. M. Helix geometry in proteins. *J. Mol. Biol.* **201**, 601-619 (1988).
2. Halkier, A., Helgaker, T., Jorgensen, P., Klopper, W. & Olsen, J. Basis-set convergence of the energy in molecular Hartree-Fock calculations. *Chem. Phys. Lett.* **302**, 437-446 (1999).
3. Halkier, A. *et al.* Basis-set convergence in correlated calculations on Ne, $N_2$, and $H_2O$. *Chem. Phys. Lett.* **286**, 243-252 (1998).
4. Rezac, J., Riley, K. E. & Hobza, P. S66: a well-balanced database of benchmark interaction energies relevant to biomolecular structures. *J. Chem. Theory Comput.* **7**, 2427-2438 (2011).
5. Khaliullin, R. Z., Cobar, E. A., Lochan, R. C., Bell, A. T. & Head-Gordon, M. Unravelling the origin of intermolecular interactions using absolutely localized molecular orbitals. *J. Phys. Chem. A* **111**, 8753-8765 (2007).
6. Morozov, A. V., Kortemme, T., Tsemekhman, K. & Baker, D. Close agreement between the orientation dependence of hydrogen bonds observed in protein structures and quantum mechanical calculations. *Proc. Natl. Acad. Sci. USA* **101**, 6946-6951 (2004).
7. Pace, C. N. Energetics of protein hydrogen bonds. *Nat. Struct. Mol. Biol.* **16**, 681-682 (2009).
8. Reed, A. E., Curtiss, L. A. & Weinhold, F. Intermolecular interactions from a natural bond orbital, donor-acceptor viewpoint. *Chem. Rev.* **88**, 899-926 (1988).
